# Detection of group a streptococcal pharyngitis by quantitative PCR

**DOI:** 10.1186/1471-2334-13-312

**Published:** 2013-07-11

**Authors:** Eileen M Dunne, Julia L Marshall, Ciara A Baker, Jayne Manning, Gena Gonis, Margaret H Danchin, Pierre R Smeesters, Catherine Satzke, Andrew C Steer

**Affiliations:** 1 Pneumococcal Research, Murdoch Childrens Research Institute, Parkville, VIC, Australia; 2 Group A Streptococcus, Murdoch Childrens Research Institute, Parkville, VIC, Australia; 3 Department of Paediatrics, The University of Melbourne, Parkville, VIC, Australia; 4 Microbiology, Department of Laboratory Services, Royal Children’s Hospital, Parkville, VIC, Australia; 5 Laboratoire de Génétique et Physiologie Bactérienne, Institut de Biologie et de Médecine Moléculaires, Faculté des Sciences, Université Libre de Bruxelles, Gosselies, Belgium; 6 Department of Microbiology and Immunology, The University of Melbourne, Parkville, VIC, Australia; 7 Centre for International Child Health, The University of Melbourne, Parkville, VIC, Australia

## Abstract

**Background:**

Group A streptococcus (GAS) is the most common bacterial cause of sore throat. School-age children bear the highest burden of GAS pharyngitis. Accurate diagnosis is difficult: the majority of sore throats are viral in origin, culture-based identification of GAS requires 24–48 hours, and up to 15% of children are asymptomatic throat carriers of GAS. The aim of this study was to develop a quantitative polymerase chain reaction (qPCR) assay for detecting GAS pharyngitis and assess its suitability for clinical diagnosis.

**Methods:**

Pharyngeal swabs were collected from children aged 3–18 years (n = 91) and adults (n = 36) located in the Melbourne area who presented with sore throat. Six candidate PCR assays were screened using a panel of reference isolates, and two of these assays, targeting *speB* and *spy1258*, were developed into qPCR assays. The qPCR assays were compared to standard culture-based methods for their ability to detect GAS pharyngitis. GAS isolates from culture positive swabs underwent *emm*-typing. Clinical data were used to calculate McIsaac scores as an indicator of disease severity.

**Results:**

Twenty-four of the 127 samples (18.9%) were culture-positive for GAS, and all were in children (26%). The *speB* qPCR had 100% sensitivity and 100% specificity compared with gold-standard culture, whereas the *spy1258* qPCR had 87% sensitivity and 100% specificity. Nine different *emm* types were found, of which *emm* 89, 3, and 28 were most common. Bacterial load as measured by qPCR correlated with culture load. There were no associations between symptom severity as indicated by McIsaac scores and GAS bacterial load.

**Conclusions:**

The *speB* qPCR displayed high sensitivity and specificity and may be a useful tool for GAS pharyngitis diagnosis and research.

## Background

Group A streptococcus (GAS; *Streptococcus pyogenes*) is the most common bacterial cause of pharyngitis. GAS pharyngitis is most common in school-age children, affecting approximately 1 in 10 children per year [1]. In addition to pain and discomfort, throat infection can lead to suppurative complications such as otitis media and peri-tonsillar abscess, and non-suppurative sequelae such as rheumatic fever. GAS pharyngitis is a costly disease to society due to medical care and absence from school. In the United States, it is estimated that GAS pharyngitis costs the community up to 500 million USD per year [2].

Although GAS pharyngitis is usually self-limiting, rapid and accurate detection is important, as early treatment with appropriate antibiotics is known to reduce symptom severity and duration, decrease transmission of the organism, and reduce the risk of acute rheumatic fever [3-6]. As most pharyngitis is viral in origin, accurate diagnosis can reduce the unnecessary use of antibiotics and potential development of antibiotic resistance [7,8]. However, accurate diagnosis of GAS pharyngitis is difficult for a number of reasons. First, diagnosis of GAS pharyngitis using clinical signs alone is unreliable; physicians miss up to 50% of GAS pharyngitis cases and identify 20-40% of non-GAS sore throat cases as requiring antibiotics [9]. A contributing factor to misdiagnosis is that clinical presentation of GAS pharyngitis is variable; for example, in a study in Egypt only 31% of children with GAS pharyngitis had purulent exudates observed on clinical examination [10]. The Centor score [11] and the McIsaac score [9] (a modified version of the Centor score that takes patient age into account) use a combination of history and examination findings to aid clinical diagnosis of GAS pharyngitis, improving sensitivity from 50% up to 85% overall and 97% in children. However, specificity remains poor (67% in children) [9]. Second, the standard procedure for laboratory detection of GAS, culture on blood agar, typically requires 24–48 hours. Third, many children are asymptomatic carriers of GAS, with the prevalence of GAS throat carriage estimated at 12% [12].

Since the 1980s, commercial rapid antigen detection tests (RADTs) have been available as a means of GAS detection. The advantage of rapid diagnostic tests is that they can be quickly performed in the physician’s office. However, although RADTs have good specificity (>95%), they often have reduced sensitivity (~85%) compared to culture [13,14]. Another method of GAS detection, polymerase chain reaction (PCR), typically has higher sensitivity (>90%) and good specificity (>95%) [15,16]. Real-time quantitative PCR (qPCR) assays provide information on bacterial cell density, which can be used to assess the limit of detection of other assays such as RADTs, and to address scientific questions such as the relationship between bacterial density and disease severity.

In this study, we screened six candidate PCR assays using reference isolates and examined the sensitivity and specificity of two qPCR assays for detecting GAS pharyngitis. We also investigated how clinical data related to GAS prevalence and bacterial load.

## Methods

### Study participants

This was a prospective observational study of patients aged 3 years and older presenting with acute sore throat to primary care over the winter/spring of 2011 and 2012 in metropolitan Melbourne (Victoria, Australia). Recruitment occurred at three suburban general practices and the emergency department of Melbourne’s major tertiary pediatric hospital (Royal Children’s Hospital). Exclusion criteria were: previous oral antibiotics within the last week or intramuscular benzathine penicillin in the last month, history of rheumatic heart disease or post streptococcal glomerulonephritis, hospitalization, immunosuppression, obvious alternate diagnosis (such as herpes gingivostomatitis or hand foot and mouth disease), language barrier or inability to give consent. Antibiotics were prescribed to patients at the discretion of the treating physician. Demographic information, clinical data and throat swabs were collected at presentation. Clinical data were used to calculate the McIsaac score for each patient [9].

### Sample collection, detection of GAS by culture, and *emm*-typing

Two throat samples were obtained using standard methods [3], rubbed together to facilitate even distribution of bacteria, and transported to the Royal Children’s Hospital laboratory within 48 h (stored at ambient temperature if processed the same day of collection and at 4°C if kept overnight). One swab was used for detection of GAS by culture as previously described [1], with streptococcal grouping performed with the Prolex Streptococcal Grouping Latex kit (Pro-Lab Diagnostics, Richmond Hill, Canada). GAS growth was scored as follows: rare (<10 β-hemolytic colonies in the first quadrant only), 1+ (≥10 in the first quadrant only), 2+ (≥10 in the first and second quadrants only), 3+ (≥10 in the first, second, and third quadrants only), and 4+ (≥10 in all four quadrants). *emm*-typing was performed as described by the Centers for Disease Control and Prevention (http://www.cdc.gov/ncidod/biotech/strep/protocol_emm-type.htm) with the following modifications: 500 nM primer concentration, and PCR cycling conditions were a 5 min activation at 95°C, followed by 30 cycles of amplification at 95°C for 15 s, 46.6°C for 30 s, and 72°C for 90 s and a final extension at 72°C for 10 min.

### PCR on reference isolates

Primer pairs shown in Table 1 were tested against a panel of reference isolates shown in Table 2, present in our culture collection or kindly provided by Prof. Roy Robins-Browne, The University of Melbourne. Bacterial DNA was extracted from fresh overnight cultures using a DNeasy Blood and Tissue kit (Qiagen, Doncaster, Australia). PCRs were performed in 25 μl reactions containing approximately 10 ng genomic DNA, 0.125 U Amplitaq Gold DNA Polymerase, 1X PCR Gold Buffer (Applied Biosystems, Mulgrave, Australia), 2.0 mM MgCl_2_, 400 nM forward and reverse primers (Sigma-Aldrich, Sydney, Australia), and 200 μM each deoxynucleoside triphosphate (Promega, Alexandria, Australia). PCR cycling conditions were an initial 5 min at 95°C step, followed by 35 amplification cycles of 95°C for 30 s, 64°C for 30 s, and 72°C for 45 s, and a final extension at 72°C for 7 min. PCR products were examined by gel electrophoresis.

**Table 1:** PCR assays selected for screening reference isolates

**Target**	Primer and probe sequences (5’-3’)*	**Product size (nt)**	**Reference**
*speB*	1F: GGTTCTGCAGGTAGCTCTCG	346	[17]
	1R: TGCCTACAACAGCACTTTGG		
	2F: CTAAACCCTTCAGCTCTTGGTACTG	77	This study
	2R: TTGATGCCTACAACAGCACTTTG		
	probe: Cy3-CGGCGCAGGCGGCTTCAAC-BHQ2		
*parE*	1F: CAACAGATGCTACGGGATTGCAC	139	[18]
	1R: GTCAGTGTGGCAGATAGCGGACG		
*spy1258*	1F: AAAGACCGCCTTAACCACCT	450	[19]
	1R: TGGCAAGGTAAACTTCTAAAGCA		
	2F: ACCTCAAATTTCCGCAACTC	141	This study
	2R: TGCTCTCAATACTGGCAAGG		
	probe: Cy3-TGGTTTCCAAGACATTGTGACCAATCA-BHQ2		
*spy1857*	1F: CCTGCACCTGACATTTCAAC	155	This study
	1R: GAAGGTATTGAAGGCCGTGT		

*probes used for quantitative PCR assays only.

**Table 2:** PCR and qPCR results for streptococcal reference isolates

	PCR assay*						qPCR assay**	
**Species and strain**	***speB*(1)**	***speB*(2)**	***parE***	***spy1258*(1)**	***spy1258*(2)**	***spy1857***	***speB***	***spy1258***
							**ct value**	**ct value**
*S. pyogenes* IGL 1	+	+	-	+	+	+	19.5	19.9
*S. pyogenes* IGL 6	+	+	-	+	+	+	21.1	20.2
*S. pyogenes* IGL 13	+	+	-	+	+	+	21.6	20.3
*S. pyogenes* IGL 165	+	+	-	+	+	+	21.2	20.4
*S. pyogenes* IGL 181	+	+	+\-	+	+	+	19.5	19.6
*S. pyogenes* ATCC BAA-572	+	+	+\-	+	+	+	21.1	20.7
*S. pyogenes* IRP 187	+	+	-	+	+	+	20.2	19.9
*S. pyogenes* 85RP187	+	+	+	+	+	+	21.1	20.0
*S. mitis* PMP933	+\-	+\-	NS	+\-	+\-	+\-	No Ct	No Ct
*S. mitis* PMP934	+\-	+\-	NS	+\-	+\-	+\-	No Ct	34.8
*S. mitis* PMP16	+\-	+\-	-	+\-	+\-	+\-	No Ct	No Ct
*S. pneumoniae* ATCC 6305	-	-	+	-	-	-	No Ct	No Ct
*S. agalactiae* ATCC 13813	-	-	NS	-	-	+	No Ct	No Ct
*S. agalactiae* GBS78	+\-	-	NS	-	+\-	+	No Ct	No Ct
*S. agalactiae* GBS79	-	-	-	+\-	+\-	+	No Ct	No Ct
*S. sanguis* NTCT7864	-	-	NS	-	-	-	No Ct	No Ct
*S. mutans* PMP935	-	-	NS	-	-	+	No Ct	No Ct

* + = strong PCR product at expected size; +/− = weak PCR product at expected size; NS = non-specific (PCR product at unexpected size and/or multiple PCR products); - = no PCR product.

** Cycle threshold (Ct) values are reported as the mean of duplicate wells containing 0.4 ng genomic DNA.

Primer and dual-labeled probe sequences for the *speB* and *spy1258* qPCR assays are shown in Table 1. qPCRs were performed on reference isolates in duplicate 25 μl reactions containing approximately 0.4 ng genomic DNA, 100 nM forward and reverse primer, 150 nM probe (Eurogentec, Seraing, Belgium), and 1X Brilliant III Ultra-Fast QPCR master mix (Agilent Technologies, Santa Clara, USA) on a Stratagene Mx3005 realtime PCR instrument with an initial activation of 95°C for 3 min followed by 35 cycles of 95°C for 20 s and 60°C for 20 s.

### qPCR validation on clinical samples

The swab used for qPCR was stored in STGG media [20] at −80°C until use. Lysis and DNA extraction from a 100 μl aliquot was performed as previously described [21]. qPCR reactions were performed in triplicate using 1 μl of DNA in each qPCR assay as described above. DNA extracted from pure cultures of *S. pyogenes* IGL 6 was used for standard curves to calculate genome equivalents/μl of GAS. Bacterial load data are reported as CFU/ml (assuming one genome per Colony Forming Unit and a GAS genome size of 1.8 Mb).

### Statistical analysis

Analyses were conducted using Prism 5.04 (GraphPad Software, Inc., La Jolla, USA). Student’s t test were used to compare normally distributed data and Mann-Whitney and Kruskal-Wallis tests used for data that did not show normal distribution. The chi-square test for trend was used to assess GAS prevalence and McIsaac scores. Spearman's rank correlation coefficient was used to examine associations between bacterial loads by qPCR and plate growth scores and bacterial loads by qPCR and McIsaac scores. McIsaac scores and plate growth scores were examined using the Pearson correlation coefficient and chi-square test for trend. P values < 0.05 were considered statistically significant.

### Ethical approval

The study was performed in accordance with the Declaration of Helsinki and was approved by the Royal Children’s Hospital Melbourne Human Research Ethics Committee HREC 31151 and 32080. Prior to enrolment in the study, informed consent was given by participants or by a parent/guardian for participants under the age of 18.

## Results

### Patient characteristics

The 127 participants included 60 females and 67 males; 91 were children and 36 were adults. Ages ranged from 3 to 72 years with a mean age of 9 y for children and 38 y for adults.

### PCR on reference isolates

The six primer pairs (Table 1) initially tested in our collection of reference streptococcal species (Table 2) targeted four GAS genes or genetic regions (*speB*, *parE*, *spy1258*, and *spy1857*). For two target genes (*speB* and *spy1258*), published primers resulted in a product size larger than recommended for qPCR, so alternative primers generating a shorter product were designed and tested. Initial qualitative PCR revealed that the *parE* assay had limited sensitivity for GAS, whereas the *spy1857* detected several non-group A streptococcal species (Table 2). *S. mitis* displayed some cross-reactivity for all assays tested. Based upon these results, two assays targeting *speB* (encoding a cysteine protease [22]) and *spy1258* (encoding a putative transcriptional regulator [19]) were selected for qPCR assay development. The optimal number of qPCR cycles was determined to be 35 to avoid false positive results with *S. mitis*, *S. sanguis* or *S. agalacticae*. Only one isolate of *S. mitis* showed faint cross-reactivity for the *spy1258* assay (Ct of 34.8; Table 2). The limit of detection for both qPCR assays was 24 genome equivalents/μl, as this corresponded to the lowest value on the standard curve that consistently resulted in a Ct value <35.

### Culture and qPCR results from clinical samples

Of the 127 throat samples analyzed, 24 (18.9%) were positive for GAS by culture. All 24 positive samples came from children; therefore, the GAS-positive proportion in this age group was 26%. A total of nine different *emm* types were identified, with *emm*89 (6 isolates), *emm*3 (5 isolates), *emm*28 (4 isolates) the most common. Other *emm* types were emm12.0 (3 isolates), *emm*1 (2 isolates), and *emm*81, *emm*75, *emm*9, and *emm*87 (1 isolate each). Two new *emm* subtypes, *emm*3.87 and *emm*12.67, were discovered.

In comparison with culture results, the *speB* qPCR had 100% sensitivity and specificity, whereas the *spy1258* qPCR had 87% sensitivity and 100% specificity (Table 3). None of three samples positive for either group C or G streptococci were positive with our qPCR assays. The three samples for which the *spy1258* qPCR gave a false negative result were from GAS type *emm*3 (two isolates) and *emm*28 (one isolate) and the bacterial plate growth scores ranged from 1+ to 3 +.

**Table 3:** GAS qPCR results in comparison to culture

**qPCR assay**	**qPCR result**	**Culture result**		% sensitivity*	% specificity*
		**+**	**-**		
*speB*	+	24	0	100 (88, 100)	100 (96, 100)
	-	0	103		
*spy1258*	+	21	0	87 (68, 96)	100 (96, 100)
	-	3	103		

* 95% confidence intervals (Wald method) shown in parentheses.

**Figure 1:**
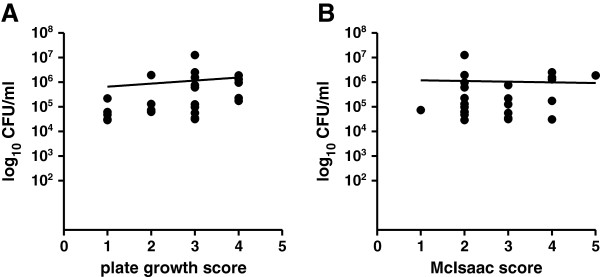
**GAS bacterial loads, plate growth, and symptom severity (McIsaac score). (A)**. GAS bacterial loads as determined by *speB* qPCR by plate growth score. P = 0.01. **(B)**. GAS bacterial loads as determined by *speB* qPCR by McIsaac score. P = 0.39. For both **A** and **B**, each data point represents CFU/ml data from one patient. Nonlinear regression curves are shown in black and P values calculated using Spearman’s correlation test.

GAS loads were then estimated using *speB* qPCR. GAS bacterial loads ranged from 2.9 × 10^4^ to 1.3 × 10^7^ CFU/ml, with a mean of 1.1 × 10^6^ CFU/ml. GAS loads by qPCR positively correlated with plate growth scores (Figure 1A; P = 0.01).

### Symptom severity

Overall, mean McIsaac scores were significantly higher for patients positive for GAS (2.7, 95% CI: 2.3, 3.1) than those who were GAS negative (1.6, 95% CI: 1.4, 1.9). This is in keeping with recently published data from the United States (Table 4; [23]). Of note, there was no association between McIsaac score and bacterial loads as determined by qPCR (Figure 1B; P = 0.39) or by plate growth score (P = 0.08).

**Table 4:** Distribution of McIsaac scores and positive GAS results

**McIsaac score**	**Total**	**GAS positive**	**Estimated risk of GAS**
	n (%)*	**n (%)**^**+**^	**(%) **[23]
≤0	25 (20)	0 (0)	8-9
1	22 (17)	1 (4)	13-14
2	40 (31)	11 (27)	23-23
3	27 (21)	6 (22)	37-37
≥4	13 (10)	6 (46)	55-56

* % of total patients with the corresponding McIsaac score.

^+^ % of patients with the corresponding McIsaac score who were GAS positive.

## Discussion

In this study, we screened six qualitative PCR assays for GAS identification and selected two candidate qPCR assays, whose ability to detect GAS pharyngitis was compared to the current gold standard, culture of a throat swab on blood agar. The *speB* qPCR assay displayed 100% sensitivity and specificity, and bacterial load data were consistent with semi-quantitative measurements of plate growth. It is unclear why *spy*1258 had lower sensitivity, as the failure to detect three GAS isolates appears unrelated to bacterial load or *emm* type. However, no internal control for PCR inhibition was used, so it is possible that inhibition may have contributed to the reduced sensitivity of the *spy*1258 assay. Although the *speB* qPCR had excellent sensitivity and specificity, this assay would require further optimization to be used as a rapid diagnostic tool given the current lengthy DNA extraction protocol (optimized to maximize DNA yields). The LightCycler PCR assay for GAS detection was developed as a diagnostic tool [16], but unlike the *speB* qPCR assay described here, it is not typically performed with a standard curve and does not provide quantitative data on bacterial loads.

Differentiation between acute GAS pharyngitis and pharyngeal carriage remains a challenge and further studies should include asymptomatic carriers. Potential differences in bacterial load between GAS carriage and GAS infection could be evaluated using *speB* qPCR in a larger, population-based study. It is likely that other differences between the carrier and infective state, such as host response or presence of virulence factors, will also be important. In this study, we did not see a correlation between symptom severity as indicated by McIsaac score and GAS bacterial load as determined by qPCR or by plate growth scores. Although variation in throat swabbing techniques can impact the ability to evaluate bacterial loads, in this study, all samples were collected in a consistent manner by two trained co-investigators. A recent report by Cohen et al. [24] suggested that heavier plate growth was associated with a trend towards higher McIsaac scores in children with pharyngitis. However, the reported P value was 0.09 and plate growth scored as either heavy (3+) or light (1+ and 2+). In another study by the same group that included asymptomatic children, throat swabs from asymptomatic carriers of GAS were less likely to have heavy plate growth than swabs from children with GAS pharyngitis [25]. The link between lower bacterial load and the carrier state should be further investigated by quantitative methods such as the *speB* qPCR. This assay may also help in assessing whether RADT-negative, culture-positive children may represent GAS carriers.

The proportion of children with sore throat with a GAS positive culture in our study (26%) is within the 15-30% range typically reported [26] and is similar to earlier studies performed in metropolitan Melbourne [1,27]. The *emm* types identified were also similar to those reported in a previous study in Melbourne [1] and are among those most common in high-income countries [28,29].

## Conclusions

This study identified *speB* qPCR as a highly sensitive and specific assay for detecting GAS in throat swabs. The assay may be useful as a diagnostic tool in the future, allowing accurate identification of patients with GAS sore throat. In addition, further investigation into the relationship between bacterial load as determined by qPCR and GAS pharyngeal infection, or carriage, is warranted.

## Competing interests

Sample collection was initially funded in part by Quidel Corporation as part of a separate project to evaluate a commercial RADT. However, the RADT project was discontinued and Quidel Corporation had no involvement with the current study.

## Authors’ contributions

EMD participated in study design, carried out qPCR, performed statistical analysis, and drafted the manuscript. JLM carried out sample collection, participated in study design, and helped draft the manuscript. CAB carried out sample collection and study coordination. JM designed and performed PCR assays and assisted in qPCR optimization. GG participated in protocol design and oversaw diagnostics by culture. MHD participated in study design and coordination. PRS and CS participated in study design and edited the manuscript. ACS conceived of the study, oversaw its design and coordination, and edited the manuscript. All authors read and approved the final manuscript.

## Pre-publication history

The pre-publication history for this paper can be accessed here:

http://www.biomedcentral.com/1471-2334/13/312/prepub
